# Therapeutically targeting type I interferon directly to XCR1+ dendritic cells reveals the role of cDC1s in anti-drug antibodies

**DOI:** 10.3389/fimmu.2023.1272055

**Published:** 2023-10-24

**Authors:** Paul Noe, Joy H. Wang, Kyu Chung, Zhiyong Cheng, Jessica J. Field, Xiaomeng Shen, Stephanie C. Casey, Christa L. Cortesio, Cinthia V. Pastuskovas, Hyewon Phee, Kristin V. Tarbell, Jackson G. Egen, Amy-Jo Casbon

**Affiliations:** ^1^ Oncology Research, Amgen Research, South San Francisco, CA, United States; ^2^ Pharmacokinetics and Drug Metabolism, Amgen Research, South San Francisco, CA, United States; ^3^ Therapeutics Discovery, Amgen Research, South San Francisco, CA, United States

**Keywords:** immunogenicity, interferon, immunotherapy, dendritic cells, conventional type I DCs, antibody IFN fusion, pharmacokinetics

## Abstract

Conventional type 1 dendritic cells (cDC1s) are superior in antigen cross-presentation and priming CD8^+^ T cell anti-tumor immunity and thus, are a target of high interest for cancer immunotherapy. Type I interferon (IFN) is a potent inducer of antigen cross-presentation, but, unfortunately, shows only modest results in the clinic given the short half-life and high toxicity of current type I IFN therapies, which limit IFN exposure in the tumor. CD8^+^ T cell immunity is dependent on IFN signaling in cDC1s and preclinical studies suggest targeting IFN directly to cDC1s may be sufficient to drive anti-tumor immunity. Here, we engineered an anti-XCR1 antibody (Ab) and IFN mutein (IFN^mut^) fusion protein (XCR1Ab-IFN^mut^) to determine whether systemic delivery could drive selective and sustained type I IFN signaling in cDC1s leading to anti-tumor activity and, in parallel, reduced systemic toxicity. We found that the XCR1Ab-IFN^mut^ fusion specifically enhanced cDC1 activation in the tumor and spleen compared to an untargeted control IFN. However, multiple treatments with the XCR1Ab-IFN^mut^ fusion resulted in robust anti-drug antibodies (ADA) and loss of drug exposure. Using other cDC1-targeting Ab-IFN^mut^ fusions, we found that localizing IFN directly to cDC1s activates their ability to promote ADA responses, regardless of the cDC1 targeting antigen. The development of ADA remains a major hurdle in immunotherapy drug development and the cellular and molecular mechanisms governing the development of ADA responses in humans is not well understood. Our results reveal a role of cDC1s in ADA generation and highlight the potential ADA challenges with targeting immunostimulatory agents to this cellular compartment.

## 1 Introduction

Immunotherapy has revolutionized the field of oncology with the development of immune checkpoint inhibitors (ICIs), which have dramatically improved survival in patients with solid tumor indications that previously showed high resistance to traditional therapies and had no treatment options (1, 2). Unfortunately, only a subset of patients achieves long-term response to ICI therapy. Mounting evidence suggests the composition of the tumor microenvironment (TME) plays a major role in anti-cancer immunity and is an important predictor to the effectiveness of immunotherapy (3). Likewise, ICI therapy is now established to be most effective in patients with a “hot” TME and patients with a “cold” TME are insensitive to ICI therapy (4, 5). Thus, a major goal in immunotherapy is to develop immunomodulatory therapeutics that can stimulate the TME and convert poorly immunogenic or “cold” tumors into a “hot” tumor.

A “hot” TME is characterized as an immune-cell rich, immunostimulatory tumor with high CD8+ Teff cells (high CD8:Treg ratio), antigen-presenting cells (APCs), inflammatory M1-polarized macrophages, and immune-stimulatory cytokines (5). One of the key cytokine signaling pathways activated in “hot” tumors is type I interferon (IFN) and, correspondingly, tumors that respond to ICI therapy typically express high levels of IFN-stimulated genes (ISGs) that are critical for sustaining antitumor immune responses (4, 6, 7). In addition, across many solid tumor human cancers, a type I IFN signature correlates to good prognosis and T cell infiltration (8) consistent with type I IFN activity promoting anti-tumor immune responses. Multiple preclinical models, using a variety of syngeneic or genetically engineered mouse tumors, have demonstrated the importance of type I IFN signaling for the generation of productive anti-tumor immune responses and the ability of therapeutically delivered type I IFN to cause tumor regression (9–12). Noteworthy, intra-tumoral injection of an agonist to localize IFN production in the tumor induced response to ICI therapy in an ICI-insensitive tumor model (8); thus, suggesting tumor-localized IFN is a promising strategy to turn “cold” tumors “hot.” While recombinant type I IFN is an approved therapy in multiple solid cancers, only modest efficacy has been observed in the clinic (13, 14). Strong evidence suggests the major limitations of approved IFN therapies are poor pharmacokinetics (PK) and dose limiting systemic toxicities that prevent robust IFN signaling on relevant immune populations within the TME and tumor draining lymph nodes.

Type I IFNs are a family of cytokines that include a single IFN-beta and 14 subtypes of IFN-alphas. Type I IFNs signal through the heterodimeric IFNAR1/2 receptor, inducing a signaling cascade that induces ~1500 interferon stimulated genes (ISGs) (15). Interferons were named for their ability to restrict (or ‘interfere’) with viral replication in vertebrate cells, which is attributed to the induction of ISG genes (16). Subsequently, these transcriptional changes lead to a coordinated and sustained anti-tumor immune response across multiple cell types (17). In addition, type I IFN is a potent adjuvant for inducing primary Ab responses (18, 19) and plays an essential role in linking innate and adaptive immunity (20). In the context of tumoral immunity, type I IFNs can inhibit tumor cell survival, suppress angiogenesis, and stimulate the activity of T, natural killer, and dendritic cells (DCs). Of interest, type I IFNs can reverse the T cell suppressive activity of multiple tumor-associated myeloid cells, including macrophages, neutrophils, monocytes, and DCs by enhancing expression of T cell co-stimulatory molecules, IL-12 production, the ability to process and cross-present tumor antigens, and secretion of T cell attractive chemokines such as CXCL9/10 (16, 21).

DCs are specialized APCs that function to prime and potentiate antigen-specific T cell responses. DCs are comprised of two subsets, cDC1 and cDC2, with distinct functions and development pathways. While both cDC1 and cDC2 cells can capture tumor antigens, the cDC1 subset is unique in its ability to efficiently phagocytose and process cell-associated antigens and present antigen-derived peptides on class I MHC molecules to CD8 T cells in a process termed cross-presentation (22). Also, cDC1s produce high amounts of Th1-differentiating cytokines, including IL-12, and thus, are superior inducers of CD8^+^ cytotoxic T cells (23). Genetic ablation of the cDC1 subset in mice dramatically reduces T cell priming to tumor antigens, T cell infiltration into tumors, and immune-mediated tumor control (11, 22, 24) emphasizing the importance of this DC subset in T cell anti-tumor immunity. In mouse syngeneic tumor models, genetic ablation of the IFN-alpha receptor (IFNAR1) in DCs demonstrated that type I IFN signaling in DCs, and particularly the cDC1 subset, is required for IFN’s anti-tumor activity (9, 11). In addition, anti-tumor efficacy was also observed in mouse tumors models following peritumoral treatment with an anti-Clec9a nanobody engineered to target an IFN mutein to cDC1s (25), suggesting IFN signaling in cDC1s is both necessary and sufficient and, notably, that delivery of a DC-targeted IFN holds promise as a cancer immunotherapy. However, the use of peritumoral injection with an almost daily treatment regimen (6 - 8 treatments in 10 days) in preclinical models limits clinical translation of this therapeutic approach. Moreover, Clec9a is expressed in pDCs in mice, but reportedly not in humans (26), questioning whether pDCs may have contributed to the anti-tumor activity observed.

Given the clinical data suggesting type I IFN plays an important role in anti-tumor immunity and response to ICI therapy and the pre-clinical data demonstrating the importance of IFN signaling specifically in the cDC1 DC subset, we developed a cDC1 specific-targeted antibody (Ab) and IFN (Ab-IFN) fusion protein to determine whether systemic administration could induce selective and sustained activation of cDC1s in the tumor and potent anti-tumor activity. We found that a single treatment with the anti-XCR1 Ab and IFN mutein (XCR1Ab-IFN^mut^) fusion specifically enhanced cDC1 activation in the tumor and spleen compared to untargeted control. However, multiple treatments with the XCR1Ab-IFN^mut^ fusion resulted in robust anti-drug antibodies (ADA) and loss of *in vivo* drug binding and activity. In contrast, ADA responses and lack of *in vivo* activity was not observed with the anti-XCR1 monoclonal Ab (mAb) alone or with the untargeted Ab-IFN fusion, demonstrating that specific targeting of an immunostimulatory agent to the cDC1s may be particularly problematic. Additionally, using other cDC1-targeting Ab-IFN^mut^ fusions, we found that targeting IFN to cDC1s activates cDC1’s ability to promote Ab generation regardless of targeting antigens, ultimately resulting in ADA response. The development of ADA remains a major hurdle in immunotherapy drug development. Our results reveal that cDC1s play a role in ADA responses, highlighting the challenge of targeting these cells with a biotherapeutic approach for cancer immunotherapy due to their recently identified function in processing and presenting antigens not only to CD8^+^ T cells, but also to CD4^+^ T cells that function to enhance humoral immunity.

## 2 Materials and methods

### 2.1 Protein production

Recombinant mouse IFN-alpha15/A (Cat# 12100-9) and mouse IFN-beta (Cat#12410) were purchased from PBL Biosciences. Anti-mouse XCR1 mAb (clone MARX10), anti-human AGP3 peptide mAb (clone 4D2), anti-mouse CD11c mAb (clone N418) and Ab-IFN-alpha15/A (Ab-IFN) fusions were made on a mIgG1-SEFL1 N297A backbone with a G3G4S linker. Molecules were cloned into pTT5 expression vector and produced similar to a process described previously (27). The fusion proteins were purified from conditioned medium using MabSelect SuRe (Cytiva) and Size Exclusion Chromatography/Superdex 200 Hiload 26/600 (GE Healthcare), with a final formulation of HBSS (pH 7.6).

### 2.2 Cell lines

RAW cells that stably express XCR1 or CD11c were generated using retroviral transduction. Briefly, GP2-293 cells (Clontech #631458) were co-transfected with a murine stem cell virus (MSCV)-based retroviral vector encoding either mouse XCR1 (NM_011798.4) or mouse CD11c (NM_0213343.3) and a vector encoding the vesicular stomatitis virus (VSV) G viral envelope protein using Lipofectamine 3000 (Thermo Fisher Scientific #L3000015) according to the manufacturer’s specifications. Viral supernatant was collected 48 h post-transfection, filtered through a 0.45 µm filter, and then directly added to low passage target cells (RAW-Lucia™ ISG Cells, Invivogen) after the addition of polybrene (10 µg/mL final concentration, EMD Millipore #TR-1003-G). Transduced cells were “spinfected” for 90 min (1200g at 32°C) and allowed to recover for 48 h. RAW cells were enriched for purity and expression of the transduced gene by fluorescence-activated cell sorting (FACS) to > 95% in the final sorted population using a FACS ARIA sorter (BD Biosciences) after staining with Abs that detect mouse XCR1 (mouse IgG2βκ anti-mouse XCR1; Biolegend 148204; clone ZET) or mouse CD11c (Armenian hamster IgG anti-mouse CD11c; Biolegend 117330; clone N418) using 5 µl of labeled Ab in 100 µl of FACS staining buffer for 30 minutes on ice.

### 2.3 Ovarian tumors

Aliquots of single cell suspensions of human dissociated ovarian tumors (serous carcinoma) were purchased from Conversant Biologics (Discovery Life Sciences) and pre-screened for cell viability (> 50%) and adequate detection of major immune cell subsets to avoid evaluating tumors that lack expression of surface markers due to a processing artifact. 2 ovarian samples were selected: OVAR1 (BTC1000-J6110002946111116MS, Stage IV) and OVAR2 (BTC1000-J6110002936091316MS, Stage III-C).

### 2.4 *In vitro* binding

RAW cells were plated at 1x10^5^ cells per well in a non-TC treated U-bottom plate (Falcon 351177) and kept at 4°C. Cells were washed with Dulbecco’s phosphate-buffered saline (D-PBS) (Corning 21-031-CV) and stained with 100 μL of Fixable Viability Dye eFluor™ 780 (1:1000 dilution, Invitrogen 65-0865-18) for 30 min. Cells were again washed once with D-PBS and then blocked for 20 minutes with 100 μL of purified rat anti-mouse CD16/CD32 Mouse Fc Block (BD 553142; clone 2.4G2) at a concentration of 8.3 μg/mL. Cells were washed with staining media (PBS + 2% heat inactivated FBS + 0.05% bovine serum albumin). Cells were then stained for 30 min with 50 μl of Ab-IFN fusion proteins (starting concentration of 100 nM and 2-fold serial dilutions) or mAbs, which served as positive controls. Cells were then washed with staining media and then stained for 30 minutes with 100 uL of allophycocyanin conjugated anti-mouse IgG secondary Ab (1:500 dilution, Jackson ImmunoRes 715-136-151). Cells were then washed once with staining media and fixed in 100 μL of 4% PFA for 15 minutes at room temperature. Cells were then resuspended in staining media and analyzed on BD FACSymphony™.

### 2.5 *In vitro* IFN activity

RAW cell lines were used to measure activity of Ab-IFN fusion proteins using the IFN-inducible secreted luciferase reporter or flow cytometry to measure endogenous markers of myeloid activation (PD-L1, CD86). Cells were cultured in RPMI-1640 + GlutaMAX™ Medium (Gibco 61870036) supplemented with 10% (v/v) heat inactivated FBS (Gibco 10082147) and Penicillin-Streptomycin (Gibco 15140122). Cells were manually lifted and seeded in 100 μL in cell culture media at 30,000 cells per well in 96-well Clear Flat Bottom TC-treated Culture Microplate (Falcon 353072) and incubated (37°C, 5% CO2) for 16-24 h. Post incubation, cells were then stimulated with a dose titration of Ab-IFN fusion proteins. Fusion proteins were diluted in cell culture media with a starting concentration of 8000 nM (2-fold concentration) followed by 14 point 5-fold serial dilutions. Initial culture media from cells were aspirated, and 100 uL of cell culture media was added along with 100 μL of stimulation media. Cells were stimulated for 16-24 h in cell incubator (37°C, 5% CO2). Post stimulation, supernatant was moved to a 96-well V-bottom plate and spun down at 500 g, 4°C, for 5 min. 20 μL of supernatant was moved to a black 96-well clear bottom plate and 50 uL of reconstituted QUANTI-Luc™ (Invivogen rep-qlc1) was added. Plate was gently tapped to mix and immediately read for luminescence (Envision^®^, Perkin Elmer).

In addition to supernatant collection, cells were dislodged for Ab staining for analysis by flow cytometry. 50 μL of 0.25% Trypsin-EDTA (Gibco, 25200056) was added to the stimulated cells and incubated at 37C, 5% CO2 for 3 minutes to dislodge cells. Post incubation of Trypsin-EDTA, 150 μL of cell culture media was added and gently pipetted to dislodge adherent cells. Cells were then moved to a 96-well non-TC treated U-bottom plate to stain with Ab for flow cytometry. Cells were washed with D-PBS (Corning 21-031-CV) and stained with 100 μL Fixable Viability Dye eFluor™ 780 (1:1000 dilution, Invitrogen 65-0865-18) in D-PBS. Cells were washed once with D-PBS and Fc blocked for 20 min with 50 μL of purified rat anti-mouse CD16/CD32 Mouse Fc Block (BD 553142) at a concentration of 16.6 μg/mL diluted in staining media (D-PBS, 2% HI FBS, 0.05% BSA). Anti-mouse CD274 (PD-L1) mAb (clone MIH5), PerCP-eFluor 710 conjugated (eBioscience 46-5982-80) was diluted in staining media at a dilution of 1:200 (2x) and 50 uL of staining mixture was added onto the cells in blocking solution for a final dilution of 1:400 (1x). Cells were stained for 30 min and subsequently washed once with staining media. Cells were then fixed in 100 μL of 4% PFA (room temperature) for 15 min. Cells were then resuspended in staining media and analyzed on BD FACSymphony™.

### 2.6 Mice

6 – 8 week old female C57BL/6 and BALB/c mice were purchased from Charles River Laboratories (Hollister, CA, United States). IFNAR1 knockout mice (B6(Cg)-Ifnar1^tm1.2Ees^/J) from Jackson Laboratories (Bar Harbor, ME, United States) and BALB/c mice (CRL) were backcrossed at least 10 times to generate BALB/c-Ifnar1^tm1.2Ees^ mice. RAG2 knockout (129S6/SvEvTac-*Rag2^tm1Fwa^*) mice were purchased from Taconic. All experimental studies were conducted under protocols approved by the Institutional Animal Care and Use Committee of Amgen (IACUC). Animals were housed at Association for Assessment and Accreditation of Laboratory Animal Care (AAALAC) International-accredited facilities (at Amgen) in ventilated micro-isolator housing on corncob bedding. All mice were maintained in pathogen-free conditions in a temperature-controlled environment with 12/12 hour light/dark cycles and received sterile pellet food and reverse osmosis-purified water *ad libitum*.

### 2.7 *In vivo* pharmacokinetics

Pharmacokinetic characterization of recombinant IFN and Ab-IFN fusion proteins were tested following a single dose intravenous bolus administration in female C57BL/6 healthy mice. Blood for plasma preparation was collected at specified timepoints via submandibular vein puncture for each serial timepoint when applicable. The collected plasma specimens were stored at approximately -70°C until transferred for subsequent analysis.

### 2.8 Plasma PK quantification of IFN and antibody IFN fusions

Commercial or in-house developed immunoassays were used to measure the drug concentrations of the Ab-IFN fusion proteins, along with recombinant mouse IFN proteins (IFN-beta and IFN-alpha). To quantitate IFN-alpha and IFN-beta recombinant proteins (all from PBL), VeriKine™ Mouse IFN Alpha ELISA Kit (PBL) and VeriKine™ Mouse IFN Beta ELISA Kit (PBL) were used respectively. For mIgG1 (SEFL1) Ab-IFN-alpha15/A fusions (Ab-IFN^WT^ and Ab-IFN^A169D^), anti-IFN-alpha mAb (clone RMMA-1, PBL) was used as a capture reagent and donkey anti-mouse IgG light chain mAb (20B07-02A09, Jackson ImmunoResearch) was used as a detection reagent. Of note, suitable immunoassays could not be developed to measure drug concentrations of Ab-IFN^L53A^ fusion proteins or the XCR1 parental mAb due to lack of adequate capture with the anti-IFN-alpha mAb or the mouse XCL1 ligand (data not shown).

### 2.9 Murine tumor models

Eight-week-old mice were injected subcutaneously on the lower right flank with cancer cell lines. 3e5 MC38 (original source unknown, not authenticated), CT26 (CRL-2639, ATCC), B16.F10 (CRL-6475, ATCC) cells were injected with a 30G insulin syringe, in 100-μL suspension, on the shaved flank of briefly sedated mice. On day 10, mice were randomized into treatment groups with tumor volumes averaging 100 mm^3^ or euthanized to harvest spleens and tumors to characterize Clec9a and XCR1expression in untreated mice. Tumor volumes were measured at least 2 times a week with a caliber using the formula, tumor volume = l (length) x w (width) x h (height).

### 2.10 Tumor treatments

Unless noted otherwise, Ab-IFN fusion proteins and/or mAbs were administered via intraperitoneal (IP) injection at a dose of 10 mg/kg (or 200 μg/mouse) starting the day of randomization. For pharmacodynamic studies to evaluate Ab-IFN fusion protein activity *ex vivo*, mice were dosed with either a single injection treatment and evaluated approximately 24 h later or a multi-dose treatment (3x per week on M, TH, M) and harvested 24 h following last treatment. For efficacy studies to evaluate anti-tumor activity and/or survival, mice were dosed every 3 – 4 days (2 times per week) until the end of study (tumor volume reached ≥1500 mm^3^).

### 2.11 *In vivo* pharmacodynamics

Tumors and spleens were harvested, and single cell suspensions were prepared. In brief, tumors were mechanically minced into small fragments and placed into an enzymatical digestion buffer containing 0.2 mg/ml Liberase TL (Roche) and 20 μg/ml DNase I (Ambion), unless otherwise noted. Tumor samples were then homogenized using a gentle MACS Dissociator (Miltenyi Biotech) and incubated at 37°C for 15 minutes on a rotator at 200 rpm. Homogenized cell preparations were passed through a 70-µm cell strainer and washed with FACS wash buffer (PBS plus 2% FBS and 0.05% BSA). Spleens were manually dissociated and filtered through a 40-µm cell strainer. Red blood cells (RBCs) in spleen samples were lysed using 1 ml of RBC lysis buffer (eBioscience) at 4°C for 5 min. 1e6 single cells were loaded into 96-well plates, washed with D-PBS and kept at 4°C. Next, cells were stained with Live/Dead™ Fixable Blue Dead Cell Stain Kit (Invitrogen L23105, 1:1,000 dilution) for 30 min, followed by a wash in D-PBS, and then blocked with purified rat anti-mouse CD16/CD32 (Clone 2.4G2, BD 553142, 1:30 dilution in BD Horizon Brilliant Stain Buffer, BD 566349) for 20 min. Cell surface staining was done for 30 minutes at 4°C with an Ab cocktail (diluted in BD Horizon Brilliant Stain Buffer, BD 566349) washed twice in FACS wash buffer, fixed for 10 min at room temperature in 4% PFA, and then stored at 4°C before evaluating on the flow cytometer. Data acquisition was performed using an LSRII or FACSymphony™ flow cytometer (BD Biosciences) and analyzed using FlowJo software (Treestar). Immune cell subsets within the samples were evaluated by first generating scatter plots to select single cells (FSC-H vs FSCH-A and SSC-H vs SSCA-A), then live cells based on negative staining for the live/dead marker and finally positive staining for CD45. Within the CD45^+^ population, several cell-specific surface markers (Abs utilized are listed in **Supplementary Methods, Table I**) were then used to evaluate immune cell subsets and immune activation.

### 2.12 Anti-drug antibody immunoassay

The presence of anti-drug antibodies (ADA) was determined against anti-XCR1 mAb and Ab-IFN fusion proteins using a homogeneous bridging MSD assay. Test molecules were labeled with biotin (Thermo Fisher) and ruthenium (MSD) to be used as capture and detection reagents respectively in the MSD assay. To analyze study samples, they were combined with a mixture of the prepared capture and detection reagents, and then capture-ADA-detection complexes were formed and captured on a streptavidin-coated MSD plate (MSD). The plate was then read for electrochemiluminescence (ECL). Pretreated study samples and Group 1 samples were included in the assay as negative controls and plasma samples spiked with a goat anti-mIgG1 Ab (Jackson Immuno Research) at 1 μg/mL was included as a positive control. The presence of ADA was compared between treatment groups by measuring assay responses (ECL values).

### 2.13 Statistical analysis

Data analysis was performed using GraphPad Prism version 9.5.1 (GraphPad Software, San Diego, CA, United States). EC50 values in this study were determined after log10 transformation of molecule concentrations using a 4-parameters nonlinear fit analysis. Group/replicate sizes are indicated in figure captions.

## 3 Results

### 3.1 Selection of XCR1 as the cDC1 target

**Figure 1:**
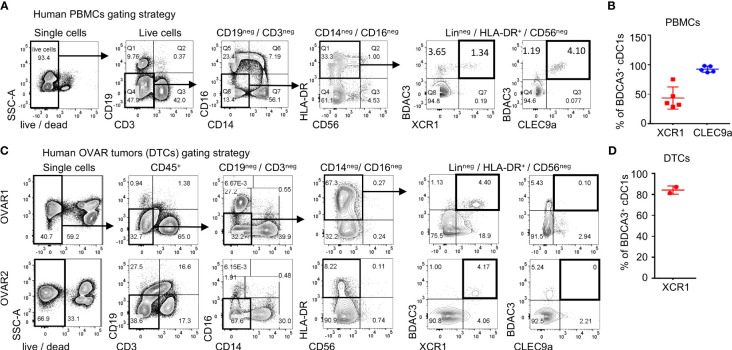
XCR1 and Clec9a expression in cDC1s in human tissues. **(A)** Gating strategy and assessment of XCR1 and Clec9a surface expression in BDCA3^+^ cDC1s from a healthy donor. **(B)** Summary of the frequency of BDCA3^+^ cDC1s in PBMCs that are XCR1^+^ or Clec9a^+^ positive across multiple healthy donors. Data shown are mean +/- SD. Pooled data is shown from 3 independent experiments with a total of 5 independent donors. **(C)** Gating strategy and assessment of XCR1 and Clec9a surface expression in BDCA3^+^ cDC1s from dissociated human ovarian tumors. **(D)** Summary of BDCA3^+^ cDC1s that were positive for XCR1 in ovarian tumors. Data shown are mean +/- SD. N=2 tumors.

To determine the surface molecule that is optimal to target cDC1s, we compared expression of Clec9a and XCR1, 2 receptors uniquely expressed on cDC1s, in human myeloid cell subsets from healthy donor peripheral mononuclear cells (PBMCs) and a small cohort of ovarian cancer tumors. Both XCR1 and Clec9a were expressed in the BDCA3^+^ cDC1 subset in PBMCs (**Figure 1A**) and did not show expression in any other immune cell subsets in whole blood (**Supplementary Figures 1A, B**). As expected, Clec9a was also not expressed in human pDCs (**Supplementary Figure 1C**). Of note, XCR1 was found to be expressed in a subset (~50%) of BDCA3^+^ cDC1s, while Clec9a was uniformly expressed in this population (**Figures 1A, B**). In ovarian tumors, XCR1 was expressed in the majority (~80%) of BDCA3^+^ cDC1s (**Figures 1C, D**), suggesting enrichment of XCR1^+^ cDC1s in tumors and possibly, an enrichment of a more mature cDC1 DC population since Clec9a and BDCA3 are reported to also be expressed in pre-DCs, while XCR1 was not (28). In contrast to XCR1, surface expression of Clec9a was undetected in the human dissociated tumor samples (**Figure 1D**), likely due to Clec9a sensitivity to clipping from enzymatic digestion based on loss of Clec9a expression in PBMCs treated with Collagenase (**Supplementary Figures 2A, B**).

**Figure 2:**
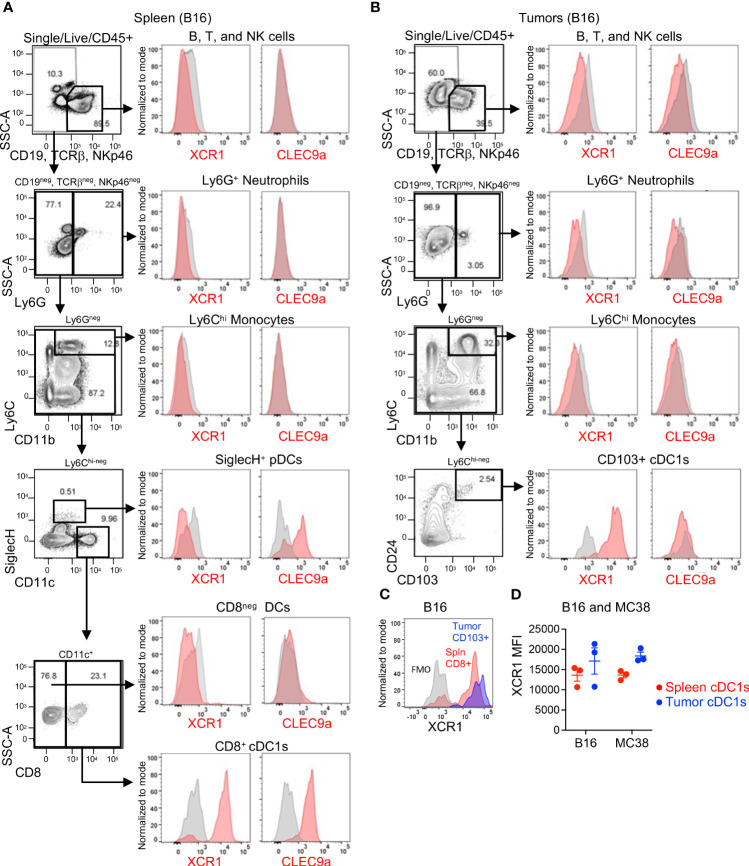
XCR1 and Clec9a expression in cDC1s in mouse tissues. XCR1 and Clec9a surface expression in non-myeloid cells (B, T, and NK cells), Neutrophils, Monocytes, pDCs, and DC subsets in **(A)** spleens and **(B)** B16 tumor-bearing mice. **(C)** Representative histogram illustrating XCR1 expression in cDC1s in spleen vs tumor from a B16-tumor bearing mouse. **(D)** Summary of XCR1 expression in cDC1s from spleens and tumors from B16 and MC38 tumor-bearing mice. XCR1 expression was evaluated by flow cytometry and measured using median fluorescent intensity (MFI). Data shown are mean +/- SD. N=3 mice/group. No statistical significance by 1-way ANOVA with Tukey’s multiple-comparison *post hoc* test. Representative data from 1 of 2 independent experiments.

Next, we evaluated XCR1 and Clec9a expression in mouse tissues using only mechanical digestion since mouse Clec9a was also found to be sensitive to enzymatic clipping (**Supplementary Figures 2C, D**), although XCR1 was not (**Supplementary Figures 2E, F**). In spleen, we evaluated XCR1 and Clec9a expression in most major immune cell subsets (B, T, and NK cells, neutrophils, monocytes, pDCs and CD11c^+^ DCs). As expected, XCR1 was only expressed in CD8^+^ cDC1s, while Clec9a was expressed in CD8^+^ cDC1s and pDCs (**Figure 2A**). In mouse tumors, XCR1 was undetected in the B/T/NK cells, neutrophils, or monocytes, but, importantly, was detected in CD103^+^ cDC1s (**Figure 2B**), establishing XCR1 as a good target for cDC1s in both human and mouse tumors. Unfortunately, Clec9a was not detected in any immune cell subsets in tumors, including CD103^+^ cDC1s (**Figure 2B**). While the mouse tumors were not enzymatically digested, we could not rule out loss of Clec9a detection due to an artifact of tissue processing since Clec9a is an endocytic receptor (29) and downregulation could also occur with release of β-actin from dying cells during tissue processing. Of interest, tumor cDC1s were approximately 90% positive for XCR1 (**Figure 2B**) and XCR1 appeared to have increased expression in cDC1s (by MFI) in tumors compared to spleen (**Figure 2C**) in both B16 and MC38 tumor-bearing mice (**Figure 2D**). Thus, based on these findings and the enrichment of XCR1 expression on tumor DCs compared to non-tumor DCs in humans and mice, XCR1 was selected as the cDC1 target.

### 3.2 Molecule design and functional characterization of cDC1-targeted Ab-IFN muteins

**Figure 3:**
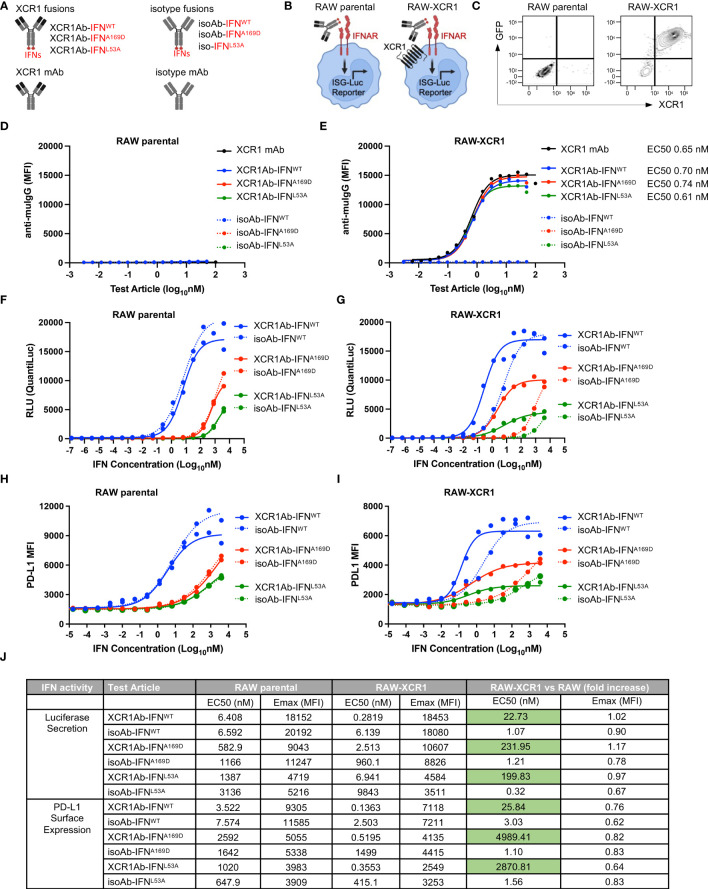
*In vitro* characterization of XCR1Ab-IFN^mut^ fusions. **(A)** Schematic illustrating Ab-IFN fusions proteins generated. **(B)** Schematic of RAW parental and RAW-XCR1 cells, illustrating these cells contain an inducible ISG-luciferase reporter and endogenously express the IFNAR1/2 receptor. **(C)** Expression of GFP and muXCR1 in RAW parental and RAW-muXCR1 cell lines. Dose response binding of molecules to **(D)** RAW parental cell lines and **(E)** RAW-XCR1 cells was evaluated by flow cytometry using a fluorescently conjugated secondary Ab to detect mouse IgG. Median fluorescent intensity (MFI) was measured. RAW parental and RAW-XCR1 cells were stimulated with XCR1Ab-IFN fusion proteins or isotype controls for 24 h. IFN activity of Ab-IFN fusion proteins was assessed by measuring luciferase secretion in the conditioned media in **(F)** RAW parental and **(G)** RAW-XCR1 cells. Relative light units (RLUs) were measured. Endogenous IFN activity of Ab-IFN fusion proteins was assessed by flow cytometry measuring PD-L1 expression in **(H)** RAW parental and **(I)** RAW-XCR1 cells. Median fluorescent intensity (MFI) was measured. **(J)** Summary of IFN activity by EC50 and Emax for luciferase and PD-L1. **(D–I)** Each experiment was performed in duplicate or triplicate with similar results.

To target type I IFN to XCR1^+^ cells, we generated anti-mouse XCR1 Ab fusions to mouse IFN-alpha isoform 15/A (referred to as XCR1Ab-IFN^WT^ fusion), which has medium to low activity compared to the other mouse IFN isoforms (30). In addition, XCR1 Ab fusions were generated with two IFN muteins, A169D and L53A (XCR1Ab-IFN^A169D^ and XCR1Ab-IFN^L53A^) (**Figure 3A**), which were selected based on conserved amino acids between mouse and human of IFN-alpha and the low activity of these human IFN mutein counterparts (31). Untargeted IFN fusion proteins (referred to as isoAb-IFN^WT^, isoAb-IFN^A169D^, and isoAb-IFN^L53A^) were also generated using a control Ab (anti-human AGP3 peptide Ab, Clone 4D2), which lacks binding in mice and served as the isotype control (**Figure 3A**). To screen the IFN activity of the Ab-IFN fusion proteins, RAW264.7 myeloid cells expressing an IFN-inducible luciferase reporter were generated to express murine XCR1, denoted RAW-XCR1 cells (**Figures 3B, C**). All Ab-IFN fusion proteins evaluated did not bind to RAW parental cells (**Figure 3D**), but XCR1Ab-IFN fusions and the XCR1 parental mAb bound to RAW-XCR1 cells with similar binding affinity (**Figure 3E**). In contrast, isoAb-IFN fusions lacked binding to RAW-XCR1 cells (**Figure 3E**).

Next, IFN activity of the fusion proteins was assessed using the luciferase reporter and cell surface expression of PD-L1, which is directly induced by type-I IFN signaling (32). Since RAW parental cells endogenously express the type I IFN-alpha heterodimer receptor (IFNAR1/2), the XCR1Ab-IFN^WT^ and the XCR1Ab-IFN^mut^ fusions showed similar activity to their matching non-targeted isoAb-IFN fusion proteins (**Figures 3F, H**), validating the IFN proteins had the same activity in the XCR1Ab-IFN and the isoAb-IFN fusion proteins. Of interest, both Ab-IFN^mut^ fusions (IFN^A169D^ and IFN^L53A^) showed a clear reduction in IFN activity, both by EC50 and maximum (Emax) activity, compared to the Ab-IFN^WT^ fusions in RAW parental cells (**Figures 3F, H**), confirming the single point mutations in mouse IFN-alpha15 reduced IFN activity. In contrast, XCR1Ab-IFN fusions showed a clear gain in activity compared to their untargeted isoAb-IFN fusions in RAW-XCR1 cells (**Figures 3G, I**). The EC50s of XCR1Ab-IFN^WT^, XCR1Ab-IFN^A169D^ and XCR1Ab-IFN^L53A^ fusion proteins were approximately 20-, 200- and 200-fold more active in RAW-XCR1 cells compared to RAW parental cells in the luciferase assay (**Figures 3G, J**) and approximately 30-, 5,000-, and 3,000-fold greater based on PD-L1 expression (**Figures 3I, J**). Notably, XCR1Ab-IFN^mut^, A169D and L53A, showed a greater gain in potency (200 to 5,000-fold) in RAW-XCR1 cells compared to RAW parental cells, while the XCR1Ab-IFN^WT^ only showed a slight gain (20 to 30-fold) (**Figures 3G, I, J**), validating the potential to increase the therapeutic window using an Ab-targeted mutein approach.

### 3.3 XCR1Ab-IFN fusions improve pharmacokinetic profile relative to wild-type IFN and specifically target IFN activity in cDC1s

**Figure 4:**
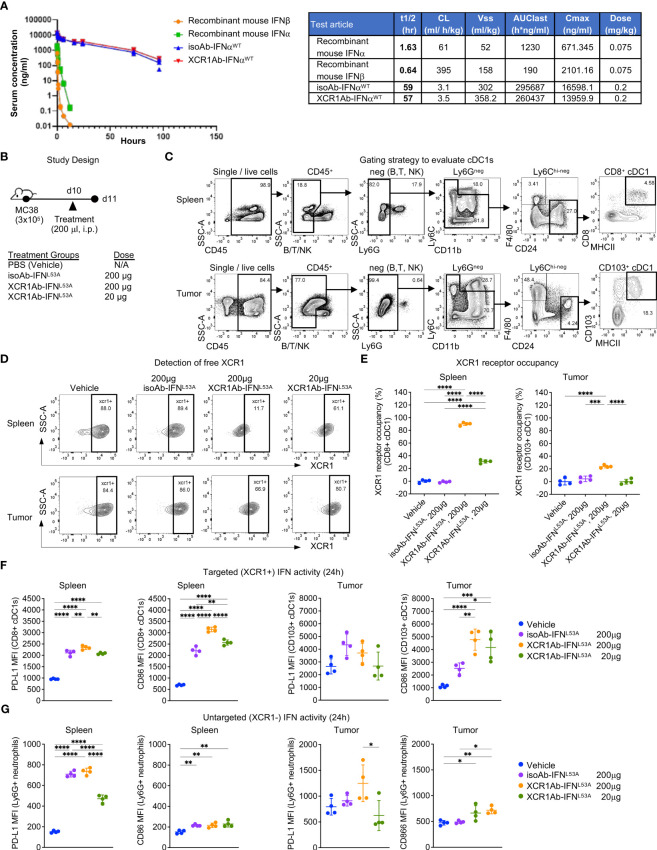
XCR1Ab-IFN fusions dramatically enhanced PK and cDC1 activation. **(A)** Pharmacokinetic profile comparison of mouse recombinant IFNs (IFN-alphaA/15 and IFN-beta) and Ab-IFN fusions of mouse IFN-alphaA/15 (IFN^WT^). Data shown are mean. N=3 mice/group. **(B)** Schematic of *in vivo* study design. **(C)** Gating strategy to assess XCR1 receptor occupancy and IFN activity in cDC1s in spleen (CD8^+^ DCs) and tumor (CD103^+^ DCs). **(D)** Detection of XCR1 surface expression using a competing PE conjugated anti-XCR1 Ab on cDC1s in spleen and tumor. **(E)** XCR1 receptor occupancy was quantified by normalizing the % of XCR1^+^ cells detected in cDC1s in treated mice to the % of XCR1^+^ cells detected in vehicle control cDC1s. PD-L1 and CD86 surface expression was evaluated by flow cytometry as a readout for IFN-induced activity in **(F)** targeted (XCR1^+^) CD8^+^ or CD103^+^ cDC1s and **(G)** untargeted (XCR1-) Ly6G^+^ neutrophils. Median fluorescent intensity (MFI) was measured. Data shown are mean +/- SD. N=4 mice/group. *P < 0.05, **P < 0.01, ***P < 0.001, ****P < 0.0001 by 1-way ANOVA with Tukey’s multiple-comparison *post hoc* test **(E–G)**.

After validating IFN targeting to XCR1+ cells was enhanced with the Ab-IFN fusions *in vitro*, we performed *in vivo* studies to measure pharmacokinetic/pharmacodynamic (PK/PD) indices. Of note, we selected the XCR1Ab-IFN^L53A^ mutein fusion and the corresponding isotype control as our proof-of-concept mutein fusions because these fusion proteins showed the most attenuated *in vitro* IFN activity (ie. based on EC50 and Emax activity), which we reasoned would result in the lowest untargeted IFN activity and toxicity *in vivo*. Immunoassays were developed to measure the drug concentration of the Ab-IFN^WT^ fusions and recombinant mouse IFN proteins (IFN-beta and IFN-alpha15), which revealed improved kinetics by increasing the half-life from 1.6 h for the recombinant IFN-alpha15 to approximate 60 h with the Ab fused IFN-alpha15 (Ab-IFN^WT^) (**Figure 4A**). Because of insufficient binding of the capturing anti-IFN Ab to the Ab-IFN^L53A^ mutein fusions, we were unable to directly measure drug concentration for this mutein.

Subsequently, we developed *ex vivo* flow cytometry PD assays to measure receptor occupancy (RO) and IFN activity of XCR1Ab-IFN fusions in cDC1s in the spleen and tumors. Mice were given a single injection of 20 μg or 200 μg (1 or 10 mg/kg) Ab-IFN fusion proteins and tissues were harvested 24 h later (**Figure 4B**). A gating strategy that utilized the restricted expression of XCR1 in CD8^+^ cDC1s in the spleen and CD103^+^ cDC1s in the tumor was adopted to evaluate XCR1 RO and IFN activity in cDC1s following treatment (**Figure 4C**). Due to the lack in availability of a non-competing anti-mouse XCR1 mAb, a fluorescently conjugated competing XCR1 Ab was used to measure the proportion of unbound or “free” XCR1 receptors on cDC1s, such that loss of XCR1 detection reflected RO of the XCR1Ab-IFN^L53A^ fusion (**Figure 4D**). In the spleen, a significant loss of XCR1 detection in cDC1s was observed from mice treated with the XCR1Ab-IFN^L53A^ fusion compared to the untargeted isotype control (**Figure 4D**) and resulted in approximately 90% and 30% RO following a 10 mg/kg and 1 mg/kg dose (**Figure 4E**). Importantly, XCR1 RO correlated to a significant increase in IFN activity in cDC1s in the spleen, measured by PD-L1 and CD86 expression, from mice treated with the XCR1Ab-IFN fusion compared to isotype control (**Figure 4F**), suggesting efficient delivery of IFN muteins to XCR1^+^ cDC1s. Moreover, a dose of 10 mg/kg isotype control fusion had slightly less CD86 expression than a dose of 1 mg/kg XCR1Ab-IFN^L53A^ fusion suggesting an approximate 10-fold gain in IFN activity in cDC1s with XCR1-targeting *in vivo*. In the tumor, XCR1 detection was also decreased in mice treated with the high dose of the XCR1Ab-IFN^L53A^ fusion (10 mg/kg) compared to the isotype control (**Figures 4D, E**), which correlated with a significant gain in IFN activity in cDC1s, based on greater PD-L1 and CD86 expression (**Figure 4F**). As expected, XCR1-negative, Ly6G^+^ neutrophils did not show a significant gain in IFN activity with the XCR1Ab-IFN^L53A^ fusion compared to the isotype control (**Figure 4G**), validating the specificity of the XCR1Ab-IFN^mut^ to increase IFN activity in cDC1s. Of note, although XCR1 RO was observed in the tumor and correlated with increased targeted IFN activity in tumor cDC1s compared to isotype control, XCR1 RO and IFN activity induced by the isotype control was much more prominent in the spleen compared to the tumor, likely due to increased drug exposure in the spleen compared to tumor.

### 3.4 Rapid loss of drug exposure of XCR1Ab-IFN fusions is dependent on IFN activity

**Figure 5:**
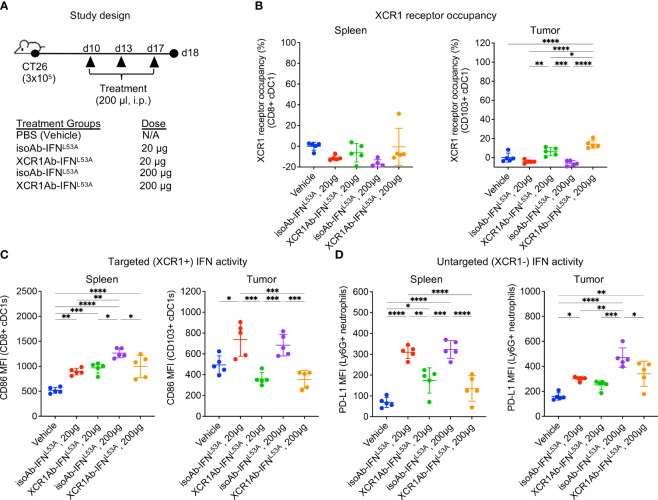
XCR1Ab-IFN^L53A^ fusion, but not isotype control, loses on-target drug binding and pharmacodynamic properties with multiple injections. **(A)** Study design to assess *in vivo* targeting of XCR1Ab-IFN^L53A^ mutein fusion to cDC1s following multiple treatments. **(B)** Free XCR1 was detected on CD8^+^ or CD103^+^ cDC1s using a competing Ab to XCR1. XCR1 receptor occupancy was quantified by normalizing the % of XCR1+ cells detected in cDC1s in treated mice to the % of XCR1^+^ cells detected in vehicle control cDC1s. IFN-induced myeloid activation (CD86 or or PD-L1 surface expression) in **(C)** targeted (XCR1^+^) cDC1s and **(D)** untargeted (XCR1-) Ly6G^+^ neutrophils was evaluated by flow cytometry. Median fluorescent intensity (MFI) was measured. Data shown are mean +/- SD. N=5 mice/group. *P < 0.05, **P < 0.01, ***P < 0.001, ****P < 0.0001 by 1-way ANOVA with Tukey’s multiple-comparison *post hoc* test **(B–D)**.

Next, we adapted a multiple dose regimen over the course of one week (**Figure 5A**) to induce continued IFN signaling in cDC1s with the goal of priming cDC1s and eliciting robust anti-tumor T cell responses. While our goal was to assess T cell infiltration using this regimen, we first repeated our *ex vivo* PD assays to confirm XCR1 RO and IFN activity was maintained. Unexpected, we did not observe significant XCR1 RO (**Figure 5B**), or a gain in IFN activity in cDC1-targeted cells in the spleen or tumor (**Figure 5C**), following 3 injections with the XCR1Ab-IFN^L53A^ fusion. In addition, in stark contrast to our results with a single treatment, IFN activity in untargeted Ly6G^+^ cells in the spleen was significantly higher with the isotype control compared to the XCR1Ab-IFN^L53A^ fusion (**Figure 5D**), suggesting a loss of drug activity and/or exposure occurs with the XCR1Ab-IFN^L53A^ fusion. Assessment of tumor volumes (**Supplementary Figures 3A, B**) at the time of tissue harvest from this PD study and from a parallel *in vivo* study designed to evaluate anti-tumor efficacy and survival (**Supplementary Figures 3C–F**) revealed no significance between the XCR1Ab-IFN^L53A^ and isoAb-IFN^L53A^ fusion treatments, further suggesting multiple injections with the XCR1Ab-IFN^L53A^ fusion was not maintaining a gain in cDC1 activation.

**Figure 6:**
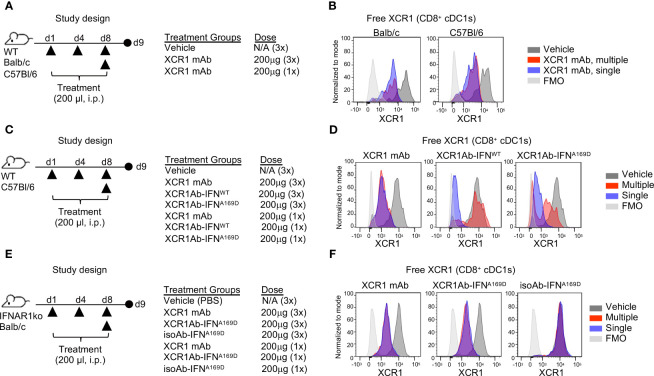
Loss of *in vivo* activity of XCR1Ab-IFN fusions is dependent on IFNAR1 signaling. **(A, C, E)** Schematic of study designs and treatment groups. **(B, D, F)** Free XCR1 expression was assessed using a PE-conjugated XCR1 detection Ab that competes with the XCR1Ab-IFN fusion proteins as a readout for receptor occupancy and *in vivo* on-target binding. Representative histograms are shown from 1 mouse from each group. N=3 mice/group.

One common explanation for loss of drug exposure is immunogenicity and the generation of anti-drug antibodies (ADA). We first evaluated whether the XCR1 mAb itself may be immunogenic and repeated our single and multi-treatment regimen in WT mice to evaluate RO (**Figure 6A**). Unlike the XCR1Ab-IFN^L53A^ fusion, the XCR1 mAb showed similar RO in the spleen following either a single or multiple treatments in WT mice from both Balb/c and C57Bl/6 backgrounds (**Figure 6B**), eliminating any strain-specific immunogenicity to the XCR1 parental Ab. Next, we hypothesized that perhaps the IFN^L53A^ mutein itself may have weak immunogenicity and targeting to cDC1s directly might enhance immunogenicity, leading to induced ADA production that was specific to this mutein. To test this hypothesis, we devised an *in vivo* experiment to assess whether other XCR1Ab-IFN fusion proteins, XCR1Ab-IFN^A169D^ and XCR1Ab-IFN^WT^, may also result in loss of drug exposure following multiple dosing and used the XCR1 mAb as the control (**Figure 6C**). Again, we used our XCR1 RO assay to measure drug coverage. Similar to the XCR1Ab-IFN^L53A^ fusion, a clear loss in RO of the XCR1Ab-IFN^WT^ and XCR1Ab-IFN^A169D^ was observed following multiple injections compared to a single injection, yet the XCR1 mAb maintained similar RO with both treatments (**Figure 6D**). These data suggest that the XCR1Ab-IFN^WT^ and XCR1Ab-IFN^A169D^ fusions were also losing drug exposure following multiple injections while the XCR1 mAb was not. Of note, the XCR1Ab-IFN^WT^ appeared to show a more significant loss in RO (ie. more free XCR1 was detected) than the XCR1Ab-IFN^A169D^ (**Figure 6D**), suggesting IFN activity might be driving the loss of drug exposure. To explore whether IFN activity was driving the loss of drug exposure of the XCR1Ab-IFN fusions, a similar *in vivo* experiment was performed using IFNAR1 knockout (IFNAR1-ko) mice, this time with the addition of the isotype control fusion (**Figure 6E**). In contrast to WT mice, we did not observe a loss of XCR1 RO with the XCR1Ab-IFN^A169D^ with multiple injections compared to a single injection in IFNAR1-ko mice (**Figure 6F**). Hence, the enhanced IFN activity in cDC1s with the XCR1Ab-IFN fusions likely plays a key role in the specific loss of drug exposure of these fusions. In summary, we found XCR1Ab-IFN fusions, but not isoAb-IFN fusions or the XCR1 mAb, were driving a robust loss of drug exposure following multiple injections in just one week.

### 3.5 Loss of cDC1-targeted Ab-IFN fusion *in vivo* activity correlates to robust ADA production

**Figure 7:**
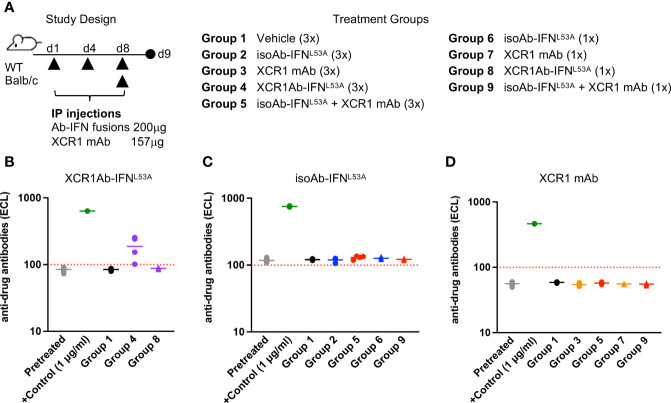
Loss of drug exposure of XCR1Ab-IFN fusions correlates to production of anti-drug antibodies. **(A)** Schematic of study design and description of treatment groups. Plasma was collected 24 h post last treatment and anti-drug Abs were measured for **(B)** XCR1Ab-IFN^L53A^, **(C)** isoAb-IFN^L53A^, and **(D)** XCR1 mAb. Each data point represents a single mouse and the horizontal line indicates the mean for the group. N=4 mice/group.

Next, we sought to determine whether the loss of drug activity of the XCR1Ab-IFN fusion proteins was the result of an immunogenic response and the development of ADA. In addition, we added a new treatment group in our study to assess whether the combination of the XCR1 mAb and the untargeted isoAb-IFN fusion could also drive this response or if it was a unique response to the XCR1Ab-IFN fusion proteins (**Figure 7A**). We found that multiple injections with the XCR1Ab-IFN^L53A^ fusion (**Figure 7B**), but not any other fusion proteins or combinations (**Figures 7C, D**), resulted in ADA production. Thus, the robust loss of drug activity, loss of drug detection, and ADA response appeared unique to treatment with the XCR1Ab-IFN fusion proteins and suggest IFN activation of cDC1s is driving the ADA response.

### 3.6 Targeting IFN to CD11c also results in rapid loss of drug exposure which is reversed in RAG2 knockout mice

To explore whether targeting IFN to a broader group of DCs, may also induce a robust ADA response or provide an alternative approach to target IFN to cDC1s, we generated a panel of CD11cAb-IFN fusion proteins, similar to the XCR1Ab-IFN fusions, and validated their ability to target and enhance IFN activity *in vitro* in CD11c^+^ RAW (RAW-CD11c) (**Supplementary Figures 4A–G**). For CD11c-targeted IFN studies, we selected the CD11cAb-IFN^A169D^ fusion, not CD11cAb-IFN^L53A^, to move forward *in vivo* because it showed the greatest gain in activity in CD11c^+^ RAW cells *in vitro*.

**Figure 8:**
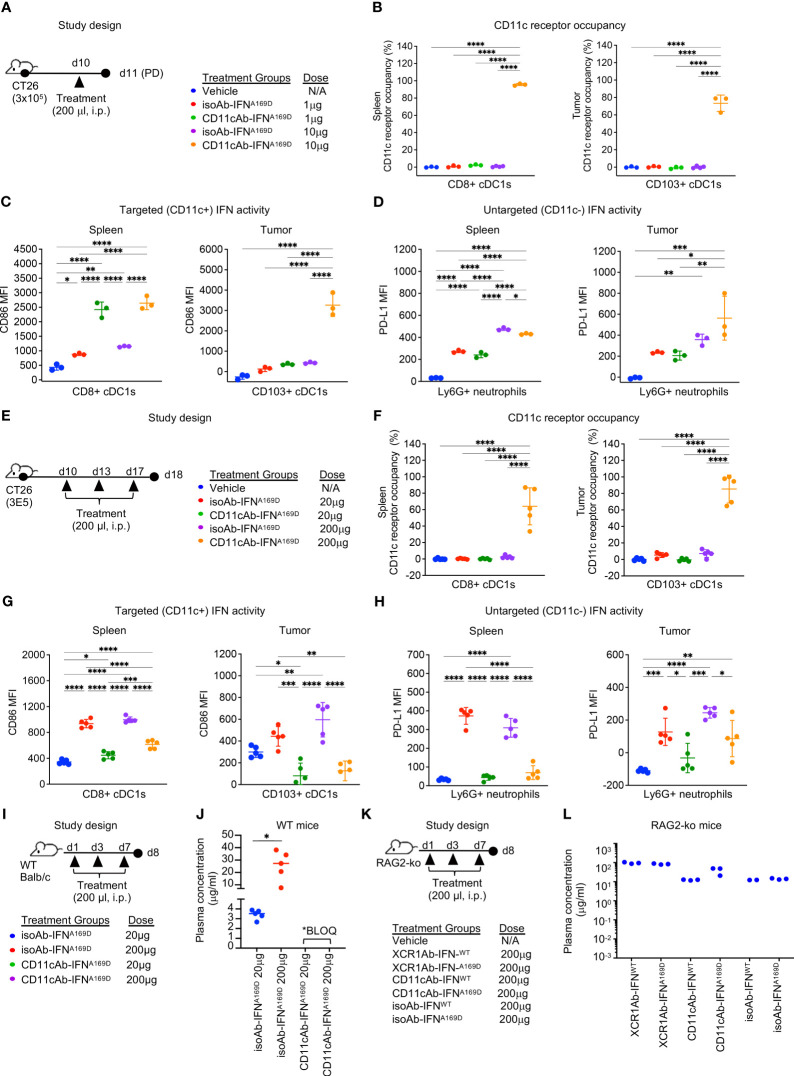
CD11c-targeted Ab-IFN fusion proteins also result in rapid loss of *in vivo* drug activity within one week following multiple injections. **(A, E, I, K)** Schematic of study designs and treatment groups. **(B, F)** Free CD11c expression was detected using a BV421-conjugated anti-CD11c (N418) Ab that competes with the CD11cAb-IFN fusion proteins by flow cytometry on cDC1s in spleen and tumor and receptor occupancy was calculated. cDC1-targeted IFN activity was assessed in CD8^+^ or CD103^+^ cDC1s **(C, G)** and untargeted IFN activity in Ly6G+ neutrophils **(D, H)** using flow cytometry. Median fluorescent intensity (MFI) was measured. Drug concentrations were measured in plasma 24 h post last dose following multiple injections in **(J)** WT mice and **(L)** RAG2 knockout (RAG2-ko) mice. **(B–D, L)** Data shown are mean +/- SD. N=3 mice/group. **(F-H, J)** Data shown are mean +/- SD. N=5 mice/group. *P < 0.05, **P < 0.01, ***P < 0.001, ****P < 0.0001 by 1-way ANOVA with Tukey’s multiple-comparison *post hoc* test.

To determine the optimal dose for the CD11cAb-IFN^A169D^ fusion to enhance IFN activity in cDC1s *in vivo*, we first performed a single injection dose response experiment (**Figure 8A**). Notably, CD11cAb-IFN^A169D^ was able to bind with specificity to cDC1s in spleen and tumor resulting in 100% CD11c RO in the spleen and ~70% CD11c RO in the tumor following a single injection with just 10 μg (0.5 mg/kg) (**Figure 8B**). In addition, CD11cAb-IFN^A169D^ induced a potent gain in IFN activity in cDC1s compared to isotype control in the spleen and the tumor at the same dose of 10 μg (**Figure 8C**). Of interest, CD11cAb-IFN^A169D^ also induced a potent gain in IFN activity in cDC1s in the spleen at the lower dose of 1 μg, which did not correlate to CD11c RO suggesting the IFN activity assay may be more sensitive and/or little CD11c RO is required to induce IFN activity in CD11c^+^ cells. As expected, CD11cAb-IFN^A169D^ and the isotype control fusion showed similar activity in untargeted Ly6G^+^ neutrophils (**Figure 8D**).

Next, we utilized our multiple injection regimen to determine whether targeting IFN to the less specific cDC1 marker, CD11c, would also result in loss of drug activity (**Figure 8E**). Of note, we also increased the concentration of the doses to anchor data to results from previous studies with the XCR1Ab-IFN fusion proteins. Multiple injections of the CD11cAb-IFN^A169D^ fusion resulted in robust loss of drug exposure as measured by lack of CD11c RO at the 20 μg dose (**Figure 8F**). In addition, loss of CD11c-targeted IFN activity in cDC1s and untargeted IFN activity in Ly6G^+^ neutrophils compared to isotype control fusions was observed at the 20 and 200 μg doses (**Figures 8G, H**). Of interest, CD11c RO was not lost at the highest dose of 200 μg, suggesting perhaps loss of drug activity could be rescued by increasing the dose; however, direct assessment of IFN activity in cells showed activity was dramatically lost even though drug binding was still observed (**Figures 8F–H**). In contrast to the CD11cAb-IFN^A169D^ fusion, the untargeted isoAb-IFN^A169D^ fusion showed a clear dose response increase in IFN activity in cDC1s in both the spleen and tumor. Likewise, assessment of tumor volumes (**Supplementary Figures 5A, B**) from this PD study and from a parallel *in vivo* efficacy study (**Supplementary Figures 5C–F**) revealed treatment with the isoAb-IFN^A169D^ fusion also led to a significant dose response decrease in tumor volumes and a significant increase in survival. Notably, treatment with the isoAb-IFN^A169D^ fusion, which lacked loss of drug exposure with the multiple injection regimen, showed superior IFN activity in cDC1s, anti-tumor activity and survival compared to the CD11cAb-IFN^A169D^ fusion (**Supplementary Figures 5E, F**). Thus, these results clearly illustrate the significant negative impact on *in vivo* activity due to the loss of drug exposure of the CD11cAb-IFN^A169D^ fusion.

To determine whether the loss in activity of the CD11cAb-IFN fusions correlated to any significant changes in PK of our fusion proteins and to further validate the role of ADA, we designed an *in vivo* study to compare drug concentrations of the Ab-IFN fusions in WT mice (**Figure 8I**) and RAG2 knockout mice that lacked Ab production (**Figure 8K**). In WT mice, only the drug concentration of the isoAb-IFN fusion was detected (**Figure 8J**). In contrast, both the CD11c-targeted IFN fusions and the isotype control IFN fusions were detected in RAG2 knockout mice (**Figure 8L**), illustrating the unique ability of type I IFN targeted to cDC1s to drive a robust humoral response and an undesired ADA response.

## 4 Discussion

### 4.1 Dual role of cDC1s to promote CD8^+^ cytotoxic T cells and CD4^+^ follicular T cells with antibody-targeted approach

Here we report a role of cDC1s in the induction of ADA. While cDC1s are well established to be efficient at cross-presenting and priming CD8^+^ T cells against cell-associated antigens, cDC1s DCs are generally regarded to have low CD4^+^ T cell activation compared to cDC2 DCs (33). However, a study using an anti-Clec9a Ab fusion with OVA (Clec9a-Ab-OVA) revealed cDC1s can be potent inducers of both CD8^+^ and CD4^+^ T cell expansion leading to a robust humoral response against both the OVA antigen and the Clec9a Ab (34). In this study, specific targeting of cDC1s with the Clec9a-Ab-OVA fusion led to a persistent *in vivo* expansion of OVA-specific CD8^+^ T cells, CD4^+^ T cells and CD4^+^ T follicular helper cells, revealing that targeting cDC1s can induce both a CD8^+^ T cell mediated response and a CD4^+^ T cell mediated humoral response. Furthermore, systemic addition of CpG as an adjuvant enhanced the cDC1-mediated CD8^+^ T cell expansion and the humoral response to the molecules. Thus, this study suggests cDC1-targeted Abs can induce an ADA response due to their biological role in stimulating CD4^+^ T cells and the humoral response. In addition, in the context of tumor immunity, a couple recent studies have also shown cDC1s to have an essential dual role in enhancing both CD8^+^ and CD4^+^ T cell activation and suggested the cDC1 activation of CD8^+^ T cells can not be separated from the dual role in activating CD4^+^ T cells (35, 36). Of interest, *Ferris et al.* (35) reveals the important role of cDC1s in anti-tumor immunity is their unique function in antigen processing and priming both CD4^+^ and CD8^+^ T cells and in orchestrating their cross-talk. Thus, shedding a new light on the role of cDC1s in tumor immunity and providing evidence that cDC1-targeted Abs may enhance ADA induction due to their dual role in stimulating both CD8^+^ and CD4^+^ T cells.

### 4.2 The development of ADA is a major hurdle in immunotherapy drug development

ADA development is a major hurdle for cancer immunotherapy (37, 38). An evaluation of the FDA’s clinical pharmacology data for 121 biological products (approved prior to 2015) reported 89% incidence of ADA, 60% impact on safety, and 49% indicated an impact on efficacy (39). There are several reasons why a biotherapeutic can induce ADA such as drug properties (e.g., non-human sequence, glycosylation, impurities, aggregation), drug pharmacokinetics, drug mechanism of action and individual patient characteristics (e.g., disease type, genetic factors, concomitant immunomodulators; probably the hardest to discern) (40). Of note, while earlier studies suggested ADA induction was due to inclusion of murine, non-human, sequences, which led the Ab field to focus on developing fully human Abs, it’s now clear that ADA induction is much more complex and many fully humanized Abs also induce ADA (41).

### 4.3 DC-targeted antibody therapies that activate antigen presentation may be prone to an ADA response

Our results suggest biotherapeutics that stimulate cDC1 (and/or CD11c^+^ DC) activation and function may be prone to induction and/or enhancement of ADA given the biology of cDC1s. Currently, there are no approved DC-targeted Ab therapies that directly stimulate DC antigen presentation to determine the clinical translation of our studies; however, two Abs (GSK2618966 and bococizumab) that failed clinical development due to ADA were recently found to have an unanticipated role in DC activation and maturation (42, 43). GSK2618966, a humanized Fc-disabled immunoglobulin G1 (IgG1) blocking Ab to the interleukin-7 receptor (IL-7R) was developed to block IL-7 signaling in T cells for the treatment of numerous autoimmune diseases (RA, type I diabetes, MS, SLE and primary Sjogren syndrome), but was discontinued during Phase I clinical studies due to its high immunogenicity (92% of subjects) and the high incidence of neutralizing Abs (42). The IL-7R, a heterodimer consisting of the IL-7Rα chain and the common γ chain, is a potent inducer of T cell proliferation, activation, and survival (44) and its expression is restricted to T cells. However, the IL-7Rα chain can also form a heterodimer with thymic stromal lymphoprotein receptor (TSLPR), which functions on myeloid cells and, importantly, plays a role in DC maturation and activation (45). Due to the enhanced immunogenicity of GSK2618966 and the role of TSLPR in activating DCs (45), GSK2618966 binding and activity in DCs was recently explored. GSK2618966 was found to bind monocyte-derived DCs and potently activate DC maturation and T cell proliferation, revealing an unexpected target-mediated immunogenicity due to the biological role of DCs (42). Another striking example is the clinical development of bococizumab, a humanized blocking Ab to proprotein convertase subtilisin/kexin type 9 (PCSK9), designed to lower low density lipoprotein cholesterol (LDL-C) for the treatment of atherosclerotic cardiovascular disease. Bococizumab, after 6 clinical trials, showed an unexpected attenuation of clinical efficacy over time and high immunogenicity, 48% developed ADA and 29% developed neutralizing ADA (46). While the mechanism for the immunogenicity of bococizumab is not well understood, recent studies suggest that the MOA of PCSK9 inhibitors is more complex than their role in lowering LDL-C and that a second, immune-mediated, mechanism may also be in play that is dependent on PCSK9 signaling in DCs (43, 47, 48). Of interest, bococizumab was found to activate CD4^+^ T cells in a PBMC assay developed to assess Ab immunogenicity and, importantly, addition of a blocking pan HLA-2 Ab resulted in loss of CD4^+^ T cell activation, suggesting bococizumab’s immunogenicity is dependent on antigen presentation by DCs (49). Thus, providing additional evidence that biotherapeutics that stimulate DC maturation (intentionally or unintentionally) may have a higher likelihood to induce ADA given their biological role in antigen presentation.

Given the significant impact in patients when ADA develop and the high cost of clinical trials, there is a need to develop ADA assays early during preclinical development and, of utmost significance, to continue monitoring ADA and effect on PK over time during clinical trials and post-marketing. For bococizumab, the phase II study only identified about 7% of ADA incidence, rather than the 48% in phase III, and if the ADA issue was properly identified in phase II, the time and cost of running 8 phase III trials might have been avoided (50). One of the reasons for the disconnect between the phase II and phase III studies was a lack of standard assays across studies, which made it difficult to summarize ADA levels. The recent development of *in vitro* assays to assess activation of CD4^+^ T cells and DCs to predict Ab immunogenicity using established controls (49, 51, 52) may provide the ability to standardize assays and prevent the development of highly immunogenic therapies from moving into the clinic.

## 5 Conclusions

In summary, we utilized a cDC1-targeted IFN mutein approach to enhance tumor immunogenicity with the goal of turning cold tumors into hot tumors; however, our study revealed a role for cDC1s in the ADA response. Specifically, our study showed that cDC1-targeted Ab-IFN fusion proteins, but not untargeted isoAb-IFN fusion proteins or the parental mAb, induced a robust ADA response within one week of treatment. ADA production correlated to loss of drug binding (receptor occupancy), *in vivo* activity and plasma concentration, which was reversed in IFNAR1 knockout and RAG2 knockout mice. Our data highlight the dual biological role of cDC1s in antigen presentation to stimulate both CD8^+^ T cells and CD4^+^ T helper cells given the specific ADA response to the cDC1-targeted Ab-IFN fusion proteins. The rapid ADA induction and loss of drug activity in the cDC1-targeted Ab-IFN fusions highlight the potential complexity in developing Ab-based therapeutics to enhance cDC1 DC tumor antigen immunogenicity due to the biological role of cDC1s and the risk of inducing ADA.

## Data availability statement

The original contributions presented in the study are included in the article/**Supplementary Material**. Further inquiries can be directed to the corresponding author.

## Ethics statement

The animal study was approved by Institutional Animal Care and Use Program. The study was conducted in accordance with the local legislation and institutional requirements.

## Author contributions

PN: Data curation, Formal Analysis, Methodology, Writing – review & editing. JW: Data curation, Formal Analysis, Writing – review & editing. KC: Data curation, Formal Analysis, Writing – review & editing, Methodology. ZC: Writing – review & editing, Data curation, Formal Analysis. JF: Writing – review & editing, Data curation, Formal Analysis. XS: Data curation, Methodology, Writing – review & editing. SC: Methodology, Writing – review & editing. CC: Data curation, Formal Analysis, Methodology, Writing – review & editing, Supervision. CP: Data curation, Writing – review & editing, Formal Analysis, Methodology, Supervision. HP: Writing – review & editing, Conceptualization. KT: Writing – review & editing, Methodology. JE: Conceptualization, Writing – review & editing. A-JC: Conceptualization, Writing – review & editing, Data curation, Formal Analysis, Investigation, Methodology, Project administration, Supervision, Validation, Writing – original draft.
