# Corrigendum: Therapeutically targeting type I interferon directly to XCR1+ dendritic cells reveals the role of cDC1s in anti-drug antibodies

**DOI:** 10.3389/fimmu.2024.1407085

**Published:** 2024-06-24

**Authors:** Paul Noe, Joy H. Wang, Kyu Chung, Zhiyong Cheng, Jessica J. Field, Xiaomeng Shen, Stephanie C. Casey, Christa L. Cortesio, Cinthia V. Pastuskovas, Hyewon Phee, Kristin V. Tarbell, Jackson G. Egen, Amy-Jo Casbon

**Affiliations:** ^1^ Oncology Research, Amgen Research, South San Francisco, CA, United States; ^2^ Pharmacokinetics and Drug Metabolism, Amgen Research, South San Francisco, CA, United States; ^3^ Therapeutics Discovery, Amgen Research, South San Francisco, CA, United States

**Keywords:** immunogenicity, interferon, immunotherapy, dendritic cells, conventional type I DCs, antibody IFN fusion, pharmacokinetics

In the published article, there was an error in the author list, and author Stephanie C. Casey was erroneously excluded. In addition, the wrong affiliation number was listed for Kyu Chung. The corrected author list appears below, along with the correct affiliations:

Paul Noe^1^, Joy H. Wang^1^, Kyu Chung^2^, Zhiyong Cheng^1^, Jessica J. Field^2^, Xiaomeng Shen^2^, Stephanie C. Casey^1^, Christa L. Cortesio^3^, Cinthia V. Pastuskovas^2^, Hyewon Phee^1^, Kristin V. Tarbell^1^, Jackson G. Egen^1^ and Amy-Jo Casbon^1^*

In the published article, there was an error in author contribution, and author Stephanie C. Casey, initials SC, was erroneously excluded. The corrected author contribution appears below:

PN: Data curation, Formal Analysis, Methodology, Writing – review & editing. JW: Data curation, Formal Analysis, Writing – review & editing. KC: Data curation, Formal Analysis, Writing – review & editing, Methodology. ZC: Writing – review & editing, Data curation, Formal Analysis. JF: Writing – review & editing, Data curation, Formal Analysis. XS: Data curation, Methodology, Writing – review & editing. SC: Methodology, Writing – review & editing. CC: Data curation, Formal Analysis, Methodology, Writing – review & editing, Supervision. CP: Data curation, Writing – review & editing, Formal Analysis, Methodology, Supervision. HP: Writing – review & editing, Conceptualization. KT: Writing – review & editing, Methodology. JE: Conceptualization, Writing – review & editing. A-JC: Conceptualization, Writing – review & editing, Data curation, Formal Analysis, Investigation, Methodology, Project administration, Supervision, Validation, Writing – original draft.

In the published article, there was an error in Supplementary Figures 3C and Supplementary Figures 5C. The study design in each figure incorrectly reports the tumor implantation date as d1 (day 1).

The correct supplemental material has been updated in the original article.

In the published article, there was an error. The formula for calculating tumor volume was incorrect.

A correction has been made to **Materials and methods**, *2.9 Murine tumor models*. This sentence previously stated:

“tumor volume = 0.52 (length *width^2^)”

The corrected sentence appears below:

“tumor volume = l (length) x w (width) x h (height)”

A correction has also been made to **Materials and methods**, *2.1 Protein production*. This sentence previously stated:

“fusions were made on a mIgG1-SEFL2 N297A backbone”

The corrected sentence appears below:

“fusions were made on a mIgG1-SEFL1 N297A backbone”

The authors apologize for these errors and state that they do not change the scientific conclusions of the article in any way. The original article has been updated.

