# Supplementary material for: Therapeutically targeting type I interferon directly to XCR1+ dendritic cells reveals the role of cDC1s in anti-drug antibodies
Source: Front Immunol. 2023 Oct 24;14:1272055. doi: 10.3389/fimmu.2023.1272055 (PMC10628189; doi:10.3389/fimmu.2023.1272055)
Supplement: Supplementary file 1 [file DataSheet_1.pdf]

Reagent	Source	Identifier	Clone
anti-human CD1c (BDCA-1)	BD biosciences	565050	F10/21A3
anti-human CD1c (BDCA-1)	Invitrogen	46-0015-42	L161
anti-human CD3	BD biosciences	564117	SP34-2
anti-human CD11c	BD biosciences	565227	B-ly6
anti-human CD14	BD biosciences	557700	M5E2
anti-human CD16	BD biosciences	563692	3G8
anti-human CD19	BD biosciences	740164	HIB19
anti-human CD45 (Ly5)	BD biosciences	562279	H130
anti-human CD56	BD biosciences	562780	NCAM16.2
anti-human CD123	BD biosciences	565050	7G3
anti-human CD141 (BDCA-3)	BD biosciences	563155	1A4
anti-human CD304 (BDCA-4)	Invitrogen	47-3049-42	TNKushoa
anti-human CD304 (BDCA-4)	Ebiosciences	61-3049-42	TNKushoa
anti-human Clec9	Biolegend	353806	8F9
anti-human Clec9	Biolegend	353804	8F9
anti-human HLA-DR	BioLegend	307616	L243
anti-human XCR1	Biolegend	372604	S15046E
anti-human Lineage Cocktail (CD3, CD14, CD16, CD19, CD20, CD56)	BD biosciences	340546	n/a
anti-mouse CD8	Biolegend	100706	53-6.7
anti-mouse CD11b	Biolegend	101226	M1/70
anti-mouse CD11c	Biolegend	117343	N418
anti-mouse CD19	Biolegend	115546	6D5
anti-mouse CD24	BD Pharmingen	563545	M1/69
anti-mouse CD45	Ebiosciences	61-0451-82	30-F11
anti-mouse CD86	Biolegend	105012	GL-1
anti-mouse CD103	Biolegend	121408	2E7
anti-mouse Clec9a	Biolegend	143504	7H11
anti-mouse Clec9a	Biolegend	143506	7H11
anti-mouse F480	Biolegend	123133	BM8
anti-mouse Ly6C	Biolegend	128041	HK1.4
anti-mouse Ly6G	BD Pharmingen	563978	IA8
anti-mouse MHCI	Biolegend	111516	KH95
anti-mouse MHCII	Biolegend	107643	M5/114.15.2
anti-mouse NKp46	Biolegend	137623	29A1.4
anti-mouse PDL1	Ebiosciences	46-5982-80	MIH5
anti-mouse Siglec-H	Biolegend	129612	551
anti-mouse TCRb	Biolegend	109234	H57-597
anti-mouse XCR1	Biolegend	148204	ZET

Supplementary Table I. Antibodies that were used in this study to evaluate myeloid cell subsets in human and mouse samples by flow cytometry.

Supplemental Figure 1

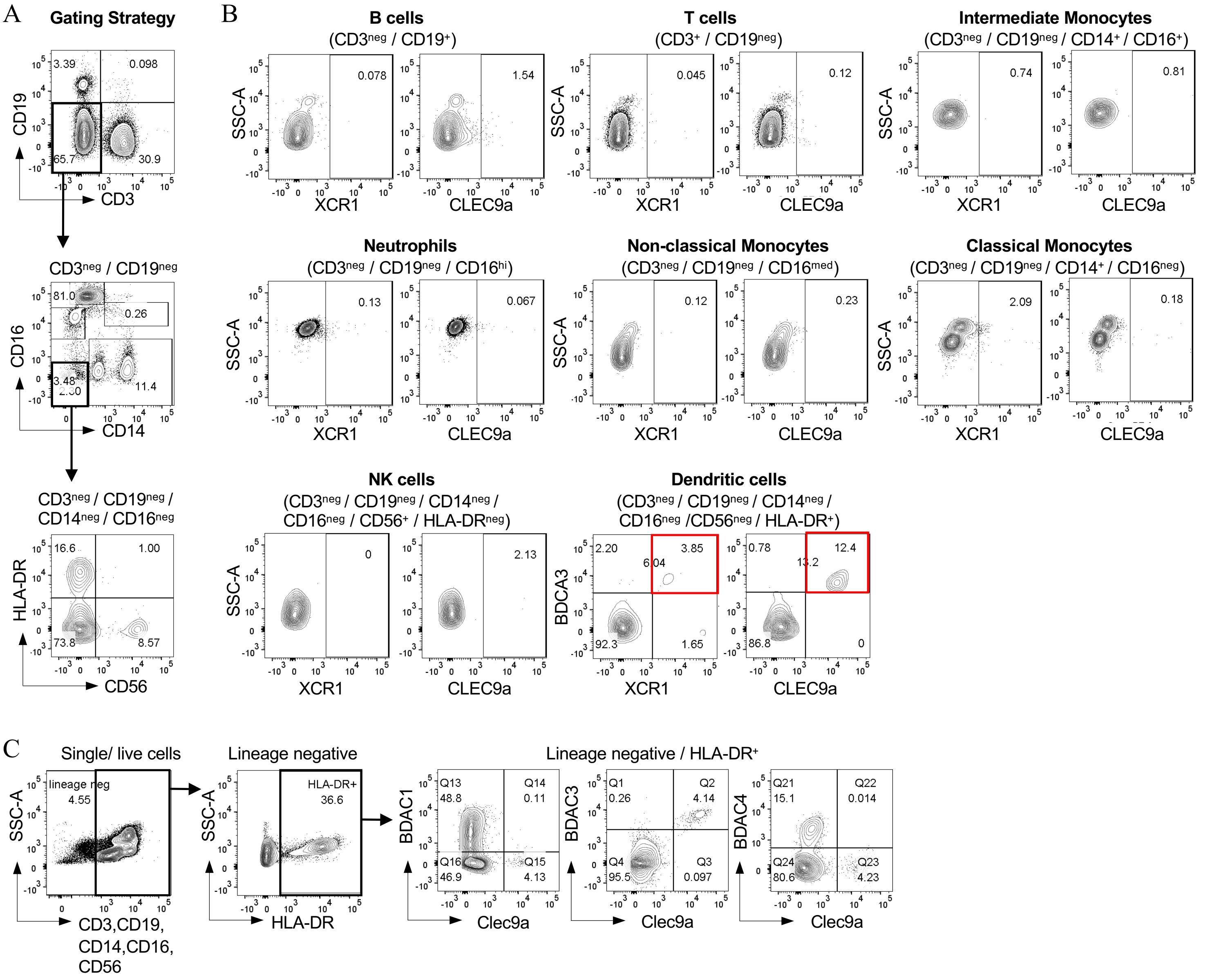

Supplementary Figure 1. XCR1 and Clec9a are specifically expressed on BDCA3+ cDC1 DCs in human blood. **(A)** Gating strategy to evaluate immune cell subsets in whole blood (Na/Citrate) from a healthy donor**. (B)** XCR1 and Clec9a expression was evaluated in multiple immune cells subsets in whole blood. Populations that showed positive expression for XCR1 or Clec9a are highlighted in the red box. **(C)** Gating strategy and expression of Clec9a in DC subsets, BDCA1+, BDCA3+ and BDCA4+ (pDCs), in PBMCs from a healthy donor.

Supplemental Figure 2

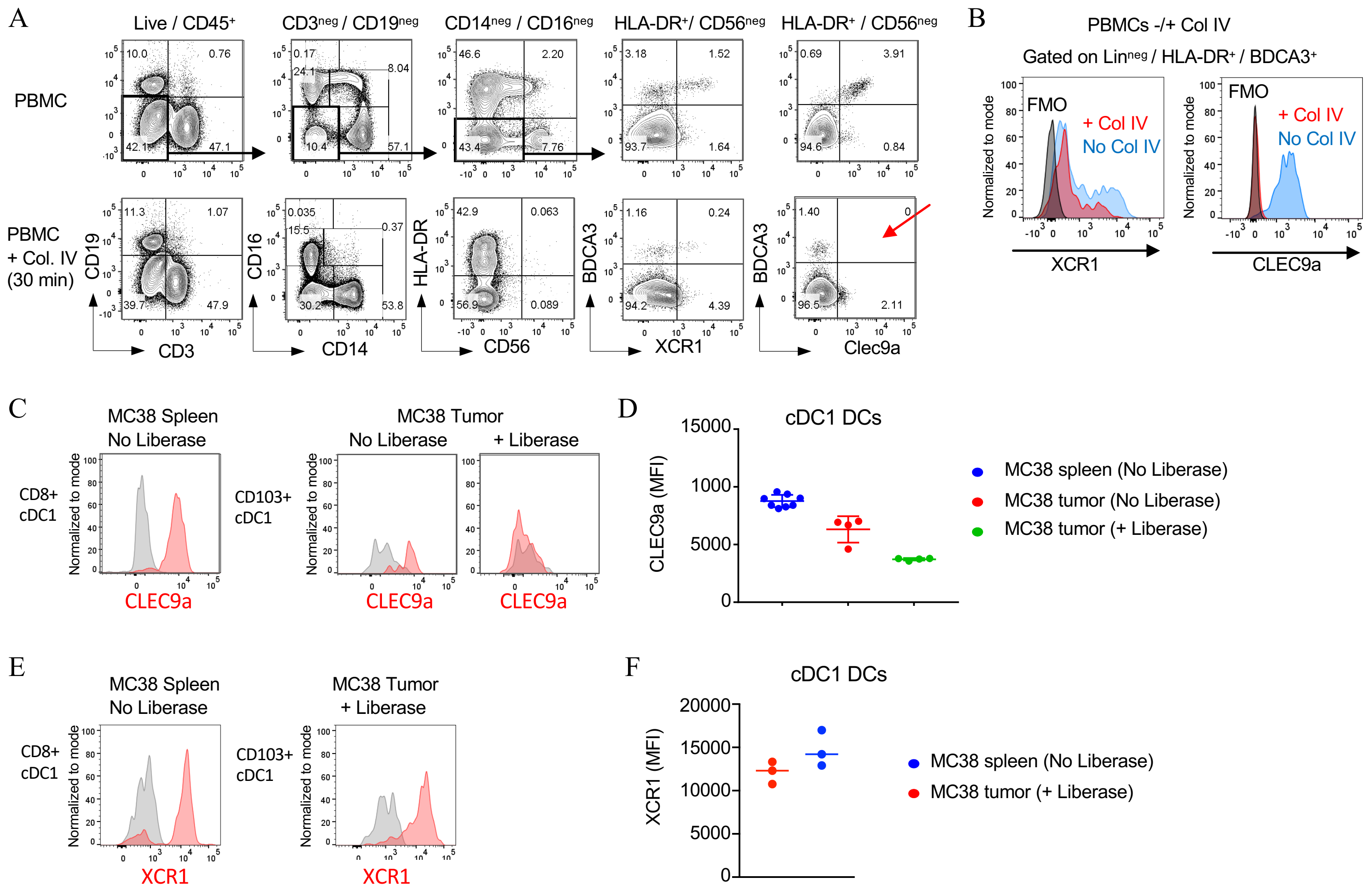

Supplementary Figure 2. Clec9a, but not XCR1, is sensitive to enzymatic cleavage in human PBMCs and mouse tissues. XCR1 and Clec9a are specifically expressed on BDCA3+ cDC1s in human PBMCs from healthy donors. **(A)** PBMCs were incubated at 37°C for 30 min in the presence or absence of Collagenase IV (0.2 mg/ml) and then evaluated for XCR1 and Clec9a expression in BDCA3+ cDC1s by flow cytometry using the gating strategy illustrated. Red arrow points to cell populations that were lost in Collagenase treated PBMCs **(B)** Comparison of XCR1 and Clec9a expression in BDCA3+ cDC1s treated with or without Collagenase IV. Spleens and tumors from MC38-tumor bearing were mechanically digested and filtered to generate single cell suspensions. Surface expression of Clec9a (**C-D**) and XCR1 (**E-F**) in mouse spleen and tumor cell suspensions.

Supplemental Figure 3

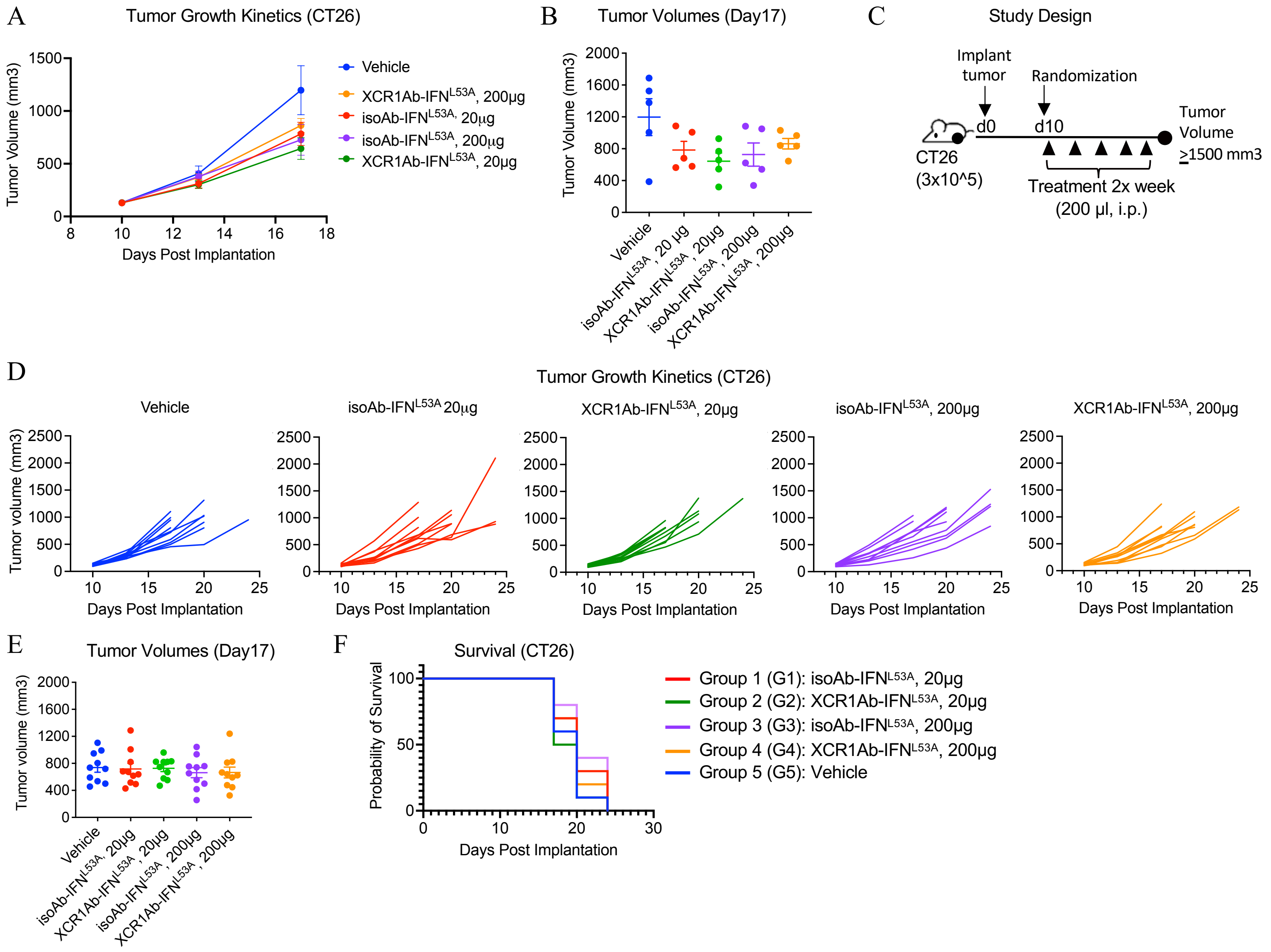

Supplementary Figure 3. *In vivo* treatment with XCR1Ab-IFN^{L53A} does not reduce tumor growth or enhance survival in the CT26 tumor model**. (A)** Tumor growth kinetics and **(B)** tumor volumes at Day 17 (time of tissue harvest) were measured from the *ex vivo* pharmacodynamic study shown in **Figure 5** in the manuscript. N=5/mice per group. **(C)** Study design to assess in vivo anti-tumor efficacy and survival in the CT26 tumor model. **(D)** Tumor growth kinetics, **(E)** tumor volumes at Day 17, and **(F)** survival. N=10/mice per group. No statistical significance in tumor volumes was observed by 1-way ANOVA with Tukey's multiple comparison adhoc test (**B and E**). No statistical significance in survival curves was observed using the Log-Rank (Mantel-Cox) test (**F**).

Supplemental Figure 4

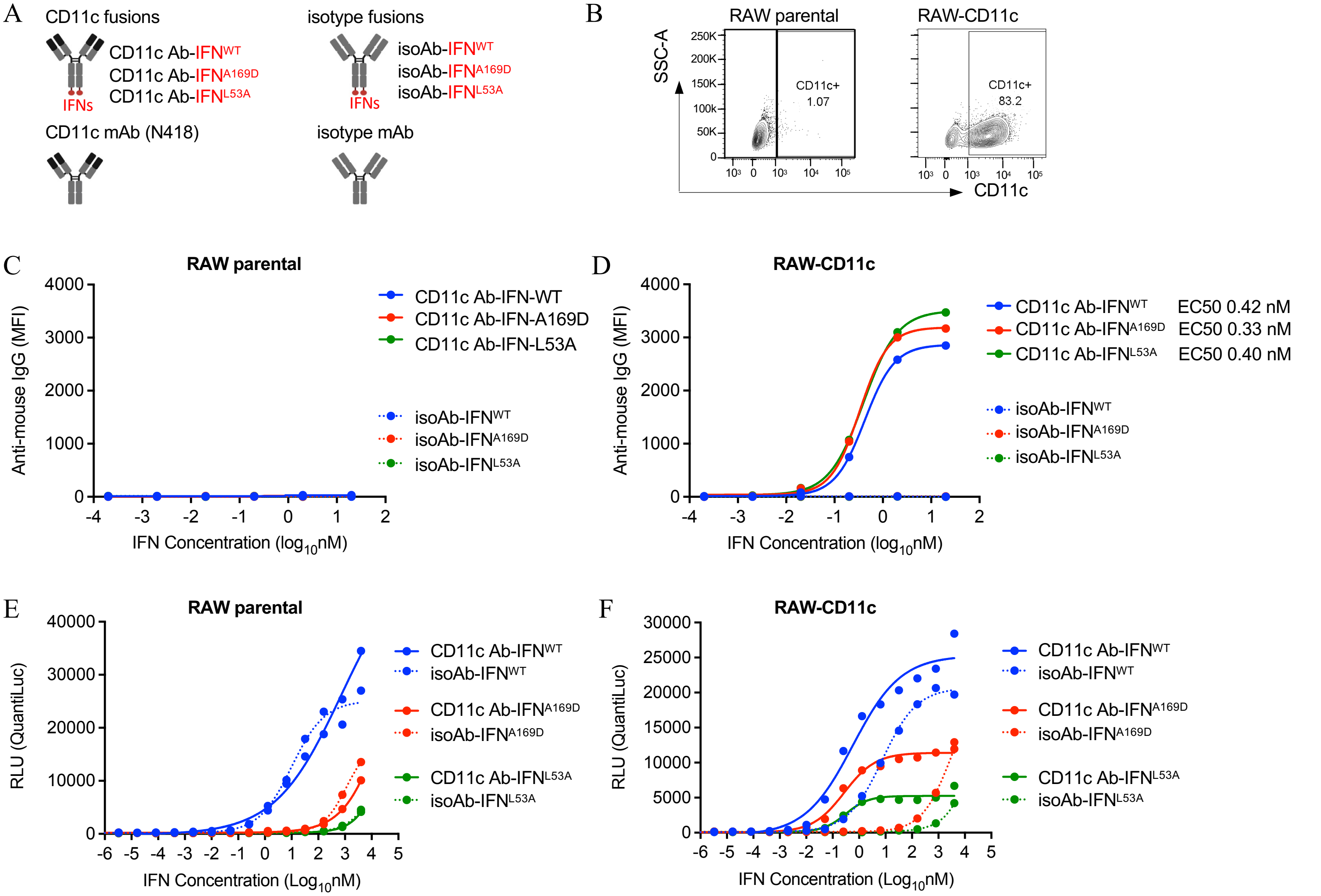

G

IFN activity	Test Article	RAW parental		RAW-CD11c		RAW-CD11c vs RAW (fold increase)	
		EC50 (nM)	Emax (MFI)	EC50 (nM)	Emax (MFI)	EC50 (nM)	Emax (MFI)
Luciferase Secretion	CD11c Ab-IFN^{WT}	736.4	34506	0.5788	28413	1272	0.82
	isoAb-IFN^{WT}	10.01	27016	7.517	20643	1.33	0.76
	CD11c Ab-IFN^{A169D}	--------	10084	0.2745	12912	--------	1.28
	isoAb-IFN^{A169D}	--------	13547	2909	11963	--------	0.88
	CD11c Ab-IFN^{L53A}	--------	4163	0.3108	6693	--------	1.61
	isoAb-IFN^{L53A}	--------	4554	---------	4215	--------	0.93

Supplementary Figure 4. *In vitro* characterization of CD11cAb-IFN fusions. **(A)** Schematic of CD11cAb-IFN fusion proteins generated and their corresponding isotype controls. **(B)** Surface expression of mouse CD11c in RAW parental and RAW-CD11c expressing cell lines. Binding of CD11cAb-IFN fusion proteins and isotype controls in **(C)** RAW parental and **(D)** RAW-CD11c cell lines. (**E**) RAW parental and (**F**) RAW-CD11c cells were cultured with CD11cAb-IFN fusions and isotype controls for 24 h, harvested and then stained for PD-L1 as a readout for IFN activity. Relative light units (RLUs) were measured in the conditioned media. **(G)** Summary of IFN activity by EC50 and Emax from **(E– F).** Representative data from 2 or 3 independent experiments with similar results.

Supplemental Figure 5

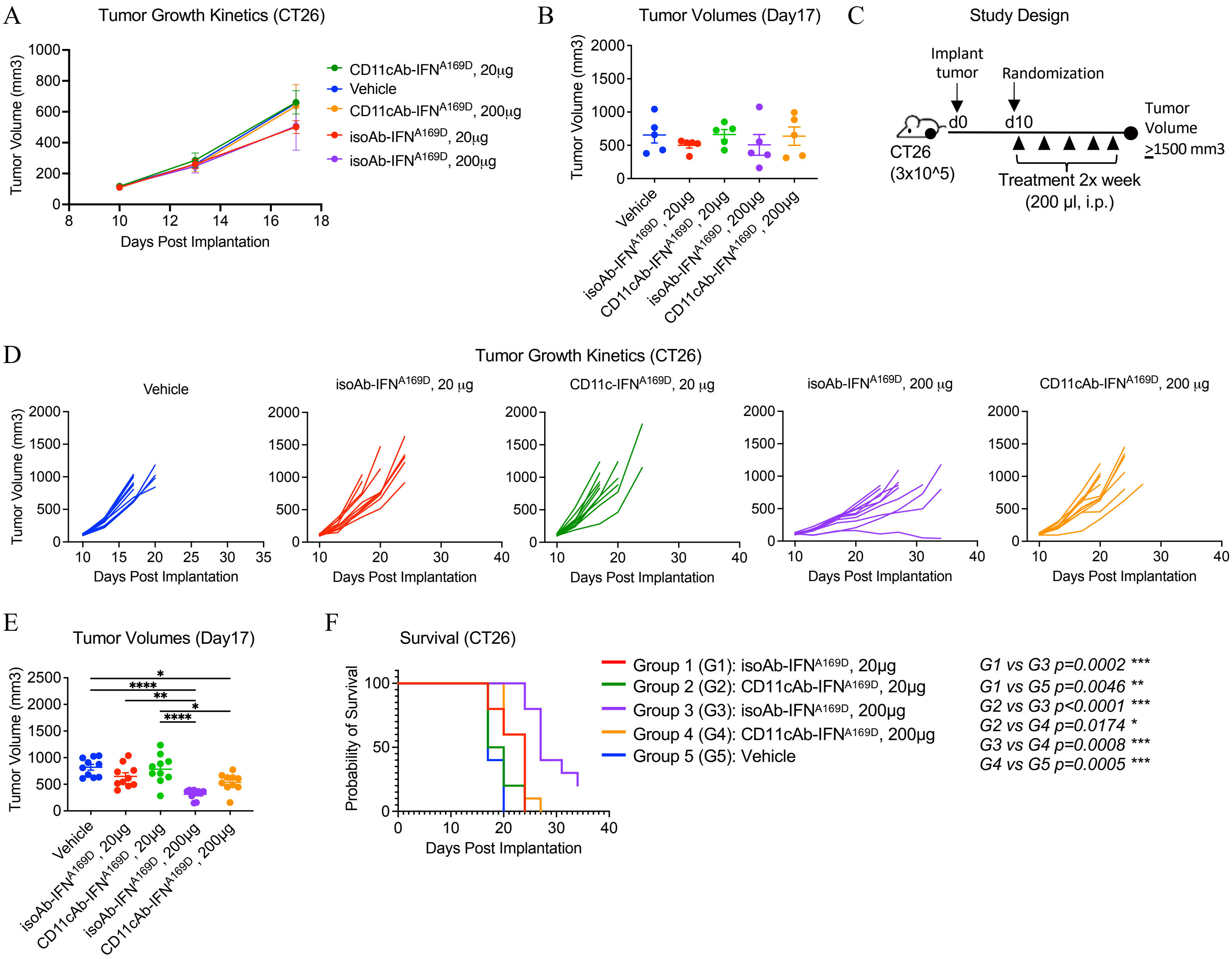

Supplementary Figure 5. Loss of drug exposure with CD11cAb-IFN^{A169D} correlates with loss of anti-tumor activity. **(A)** Tumor growth kinetics and **(B)** tumor volumes at Day 17 (time of tissue harvest) were measured from the *ex vivo* pharmacodynamic study shown in **Figure 8** in the manuscript. Data shown are mean -/+ SEM. N=5/mice per group. **(C)** Study design to assess in vivo anti-tumor efficacy and survival in the CT26 tumor model. **(D)** Tumor growth kinetics, **(E)** tumor volumes at Day 17, **(F)** and survival. Data shown are mean -/+ SEM. N=10/mice per group. Tumor volumes were compared using 1-way ANOVA with Tukey's multiple-comparison post hoc test. *$P < 0.05$, **$P < 0.01$, ***$P < 0.001$, ****$P < 0.0001$ (**B and E**). Survival curves were compared using the Log-Rank (Mantel-Cox) test. *$P < 0.05$, **$P < 0.01$, ***$P < 0.001$ (**F**).
